# Natural history of respiratory muscle strength in spinal muscular atrophy: a prospective national cohort study

**DOI:** 10.1186/s13023-022-02227-7

**Published:** 2022-02-21

**Authors:** Esther S. Veldhoen, Camiel A. Wijngaarde, Erik H. J. Hulzebos, Roelie M. Wösten-van Asperen, Renske I. Wadman, Ruben P. A. van Eijk, Fay Lynn Asselman, Marloes Stam, Louise A. M. Otto, Inge Cuppen, Feline E. V. Scheijmans, Laura P. Verweij-van den Oudenrijn, Bart Bartels, Michael A. Gaytant, Cornelis K. van der Ent, W. Ludo van der Pol

**Affiliations:** 1 Department of Pediatric Intensive Care, University Medical Center Utrecht, Wilhelmina Children’s Hospital, Utrecht University, PO box 85090, 3508 AB Utrecht, The Netherlands; 2 Department of Neurology, UMC Utrecht Brain Center, University Medical Center Utrecht, Utrecht University, Utrecht, The Netherlands; 3 Child Development and Exercise Center, Wilhelmina Children’s Hospital, University Medical Center Utrecht, Utrecht University, Utrecht, The Netherlands; 4 Biostatistics and Research Support, Julius Center for Health Sciences and Primary Care, University Medical Center Utrecht, Utrecht University, Utrecht, The Netherlands; 5 Department of Pulmonology, Center of Home Mechanical Ventilation, University Medical Center Utrecht, Utrecht University, Utrecht, The Netherlands; 6 Department of Pediatric Pulmonology, Wilhelmina Children’s Hospital, Member of ERN-LUNG, University Medical Center Utrecht, Utrecht, The Netherlands

**Keywords:** Neuromuscular, Spinal muscular atrophy, Respiratory muscle strength, Lung function, Natural history

## Abstract

**Background:**

Respiratory complications are the most important cause of morbidity and mortality in spinal muscular atrophy (SMA). Respiratory muscle weakness results in impaired cough, recurrent respiratory tract infections and eventually can cause respiratory failure. We assessed longitudinal patterns of respiratory muscle strength in a national cohort of treatment-naïve children and adults with SMA, hypothesizing a continued decline throughout life.

**Methods:**

We measured maximal expiratory and inspiratory pressure (PE_max_ and PI_max_), Sniff Nasal inspiratory pressure (SNIP), peak expiratory flow (PEF), and peak cough flow (PCF) in treatment-naïve patients with SMA. We used mixed-models to analyze natural history patterns.

**Results:**

We included 2172 measurements of respiratory muscle function from 80 treatment-naïve patients with SMA types 1c-3b. All outcomes were lower in the more severe phenotypes. Significant differences in PEF were present between SMA types from early ages onwards. PEF decline was linear (1–2%/year). PEF reached values below 80% during early childhood in types 1c-2, and during adolescence in type 3a. PE_max_ and PI_max_ were severely lowered in most patients throughout life, with PE_max_ values abnormally low (i.e. < 80 cmH_2_O) in virtually all patients. The PE_max_/PI_max_ ratio was < 1 throughout life in all SMA types, indicating that expiratory muscles were most affected. All but SMA type 3b patients had a lowered PCF. Patients with types 2b and 3a had PCF levels between 160 and 270 L/min, those with type 2a around 160 L/min and patients with type 1c well below 160 L/min. Finally, SNIP was low in nearly all patients, most pronounced in more severely affected patients.

**Conclusions:**

There are clear differences in respiratory muscle strength and its progressive decline between SMA types. We observed lower outcomes in more severe SMA types. Particularly PEF may be a suitable outcome measure for the follow-up of respiratory strength in patients with SMA. PEF declines in a rather linear pattern in all SMA types, with clear differences at baseline. These natural history data may serve as a reference for longer-term treatment efficacy assessments.

**Supplementary Information:**

The online version contains supplementary material available at 10.1186/s13023-022-02227-7.

## Background

Spinal muscular atrophy (SMA) is a severe neuromuscular disease (NMD) caused by deficiency of survival motor neuron (SMN) protein, due to homozygous loss of *SMN1* gene function. SMA demonstrates a broad range in clinical disease severity, which is reflected by the distinction of 4 types in the clinical classifications system [1, 2]. Improved understanding of the natural history of SMA has facilitated improvements of standards for supportive care and clinical trial design [1, 3–6]. Respiratory complications, such as hypoventilation and impaired secretion clearance, are the most important cause of morbidity and mortality in SMA [1, 2] but respiratory outcome measures have not yet been used as primary outcomes in clinical trials. This is, at least partially, caused by a lack of reference data [7, 8].

Respiratory muscle weakness in SMA is characterized by a rather unique pattern with predominant weakness of (mainly expiratory) intercostal muscles and relative sparing of (inspiratory) diaphragm function [9, 10]. Respiratory muscle weakness is associated with decreased pulmonary compliance, lung underdevelopment, decreased ability to cough, and it may ultimately lead to respiratory failure [11].

Improved insights into the natural history of respiratory muscle strength could guide therapeutic management [12], improve timing of supportive care [1], and facilitate its use as an outcome measure for longer-term follow-up of patients or treatment efficacy assessments [2, 7, 13, 14]. Tests of respiratory muscle strength may detect respiratory insufficiency earlier than more frequently used measurements of expiratory lung function [e.g. forced vital capacity (FVC)]. Longitudinal studies on the decline of respiratory muscle strength have been performed in other NMDs but not in SMA [15]. Therefore, we studied the natural history of respiratory muscle strength and assessed differences between SMA types in a large, population-based, treatment-naïve cohort of SMA patients.

## Methods

### Patient characteristics and general procedures

Patients participated in a prospective clinical cohort study on SMA. We captured patient characteristics using standardized questionnaires and physical examinations, including motor function assessments and lung function tests, as described previously [8, 16, 17]. We used patient data obtained prior to participation in a clinical trial or treatment with SMN protein augmenting drugs (i.e., ‘treatment-naïve’). The local Medical Ethical Committee approved this study (09-307/NL29692.041.09) and informed consent was obtained from all participants and/or their parents in case of minors. The reporting of this study conforms to the STROBE statement [18]. Homozygous loss of *SMN1* function and *SMN2* copy number were determined using multiplex ligation-dependent probe amplification (MLPA; SALSA kit P021-B1-01, MRC-Holland) [16]. Using the SMA classification system, we distinguished different SMA types as described previously (Additional File 1) [2, 6, 17, 19].

### Respiratory muscle strength tests

We used respiratory muscle strength data of included patients collected during regular visits to the outpatient departments of pulmonology and the Center for Home Mechanical Ventilation at our hospital. Not all tests were performed at each visit. Data were not collected during hospital admissions, to prevent inclusion of measurements that are influenced by, for example, the presence of respiratory tract infections. For this study we used tests on expiratory strength [maximal expiratory pressure (PE_max_), peak expiratory flow (PEF), peak cough flow (PCF)] and inspiratory strength [maximal inspiratory pressure (PI_max_), sniff nasal inspiratory pressure (SNIP)]. All measurements were unassisted, i.e. not following manual compression or lung volume recruitment with frog-breathing, air stacking or mechanical insufflation–exsufflation.

PE_max_ and PI_max_ are non-invasive tests for the direct measurement of strength of expiratory and inspiratory muscles [15]. We measured PE_max_ and PI_max_ from total lung capacity and residual volume respectively, using the Geratherm Spirostik®. A nose-clip and flanged mouthpiece were used. Air leakage was prevented by a technician holding the lips. In some cases an oronasal mask was used. At least 5 repeated attempts were made. We recorded largest pressures and compared to the reference values provided by Wilson [20]. We calculated the PE_max_/PI_max_ ratio to assess the relative impairment of expiratory versus inspiratory muscles [21, 22]. SNIP is nasal pressure measured during a maximal sniff. It is a simple test of inspiratory muscle strength. We measured SNIP in both nostrils using the Micro Medical MicroRPM®. Maximal nasal pressure during at least 5 sniffs, performed from Functional Residual Capacity, was compared to reference values [23, 24]. Finally we measured maximal flow during expiration and cough: PEF and PCF. We obtained PEF values from flow-volume curve data as the maximal expiratory flow achieved from forced expiration following maximal lung inspiration, using the Geratherm Spirostik® spirometer. We recorded the largest outcome from at least 3 qualitatively acceptable attempts. We reported absolute PEF values and standardized values [25, 26]. We measured PCF by maximal cough using both spirometry (Geratherm Spirostik®) and a peak flow meter (Assess®, PT-medical). We recorded the best outcome of 3 qualitatively acceptable attempts. Outcomes were reported as absolute values and compared to reference values [27, 28].

We measured all outcomes according to international guidelines [29], with patients in sitting position, without wearing corsets or braces. We used strong verbal encouragement and visual feedback to achieve maximal and reproducible test results. A resting period between tests prevented a significant influence of fatigability. All tests were performed by a small team of experienced professionals. We reported some outcomes as standardized values, i.e. as a percentage of the predicted value for age, height, weight, and sex. It is important to recognize that measuring height in SMA patients can be challenging. Tape-measured arm span was used preferably as a surrogate measure in patients unable to stand [8, 30].

### Statistical analysis

We performed longitudinal analyses of PE_max_, PI_max_, PE_max_/PI_max_ ratio, PEF and PCF. We used a cross-sectional analyses to assess the differences in SNIP outcomes between SMA types. A longitudinal analysis was hampered due to a too limited number of observations.

For the longitudinal analyses we used all available measurements and hypothesized progressive worsening of respiratory muscle strength over time, depending on SMA type. As it was unlikely that the longitudinal patterns were completely linear, we used non-linear analyses. We fitted smoothed B-spline models with 3 knots, in which polynomial continuous regression lines were computed in-between knots [31]. For PEF we additionally assessed the longitudinal pattern with a linear mixed-effects model (LMM). The model contained age, SMA type and an interaction term of these two predictors as fixed factors. Dependency in data due to repeated measures was accounted for by a random intercept per individual. A random slope for age was added to assess differences in rates of decline between patients (as a measure of disease heterogeneity or between-patient slope variability). We evaluated whether the rate of decline over age was significantly different between SMA types using a likelihood ratio test. For cross-sectional analysis of SNIP the first measurement of all patients was used. For the cross-sectional comparisons between SMA types we hypothesized that patients with milder SMA types would be less affected. As the assumptions of normality were met, a one-way ANOVA was used for comparisons between SMA types. A possible trend of increasing respiratory muscle strength with milder SMA types was assessed using the Jonckheere-Terpstra trend test.

## Results

### Demographics

We included 80 patients with genetically confirmed SMA types 1c–3b in this study. Ages at measurements ranged from 4.1 to 66.6 years. Baseline characteristics are shown in Table 1.

**Table 1:** Baseline characteristics

	Total number (n)	SMA type 1c (*n*)	SMA type 2a (*n*)	SMA type 2b (*n*)	SMA type 3a (*n*)	SMA type 3b (*n*)
*Participants*						
Patients	80	6	32	22	16	4
Female gender	52	3	20	14	12	3
*SMN2* copies						
2	4	1	1	1	1	–
3	66	5	29	18	12	2
4	10	–	2	3	3	2
*Tests of respiratory muscle strength*						
*Peak expiratory flow (PEF)*						
Patients	79	6	31	22	16	4
Tests	651	67	297	156	114	17
Follow-up (years) [IQR]	6.7 [1.2–12]	6.8 [3.5–8.2]	7.3 [1.3–12.2]	6.2 [1.2–11.8]	2.1 [0.4–9.8]	11.1 [7.7–15.3]
*Peak cough flow (PCF)*						
Patients	61	4	27	19	9	2
Tests	288	27	144	76	35	6
Follow-up (years) [IQR]	3.6 [0.3–8.1]	6.6 [4.7–8.8]	5.6 [0.8–7.9]	3.4 [0.3–8.3]	0.9 [0.0–2.2]	2.0 [1.0–3.0]
*Maximum expiratory pressure (PE)*_max_						
Patients	75	6	28	22	15	4
Tests	586	59	261	148	102	16
Follow-up (years) [IQR]	5.8 [1.1–10.2]	4.4 [1.8–7.1]	6.1 [1.5–11.2]	6.2 [1.0–11.5]	3.0 [0.4–9.2]	7.3 [4.9–9.6]
*Maximum inspiratory pressure (PI)*_max_						
Patients	76	6	28	22	16	4
Tests	590	60	263	148	103	16
Follow-up (years) [IQR]	6.3 [1.1–10.6]	6.7 [3.5–7.6]	6.6 [1.5–13.0]	6.2 [1.0–11.5]	2.5 [0–9.2]	7.3 [4.9–9.6]
*PE*_max_*/PI*_max_ *ratio*						
Patients	75	6	28	22	15	4
Tests	582	57	259	147	103	16
Follow-up (years) [(IQR]	5.8 [1.1–10.2]	4.4 [1.8–7.1]	6.1 [1.5–11.2]	6.2 [1.0–11.5]	3.0 [0.4–9.2]	7.3 [4.9–9.6]
*Sniff inspiratory pressure (SNIP)*						
Patients	57	3	22	19	11	2

*IQR* interquartile range, *n* number, *PCF* peak cough flow, *PEF* peak expiratory flow, *PE*_*max*_ maximal expiratory pressure, *PI*_*max*_ maximal inspiratory pressure, *SNIP* sniff nasal inspiratory pressure

### Peak expiratory flow (PEF)

**Fig. 1:**
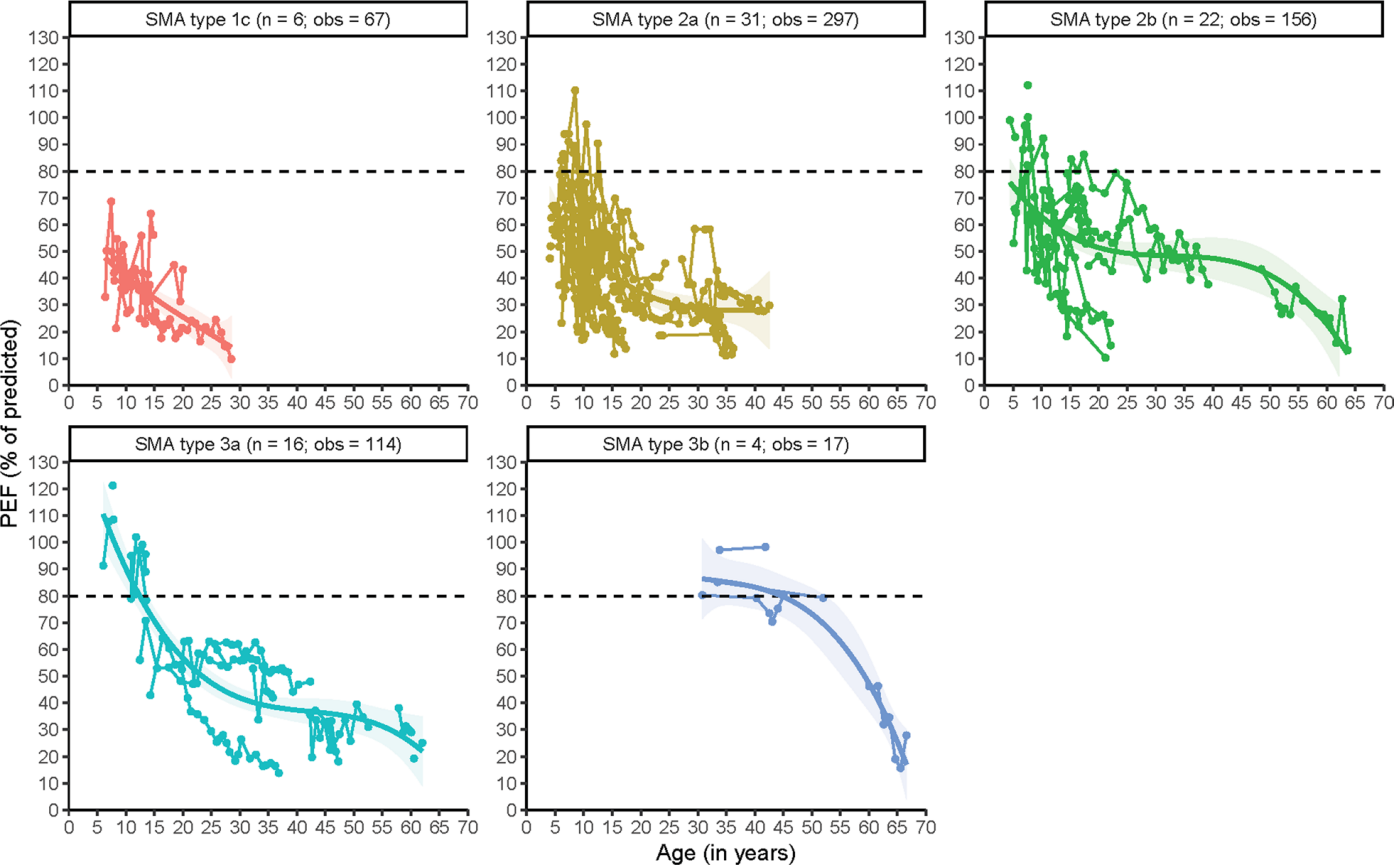
Longitudinal patterns of peak expiratory flow (PEF) (in % of predicted) in different SMA types. n = number of patients; obs = number of observations. The horizontal line at 80% of predicted PEF represents the lower limit of the normal range

We analyzed 651 longitudinal measurements of PEF from 79 patients (Table 1). At baseline, PEF values differed significantly between SMA types (Fig. 1), i.e. 49%, 73%, 87% and 96% in SMA types 1c, 2a, 2b and 3a, respectively. The estimate for patients with SMA type 3b is unreliable, due to a limited number of observations (Table 1). PEF decline to values < 80% was observed in early childhood in SMA types 1c–2b, but not until adolescence or early adulthood in type 3a. In our linear analyses the average annual rates of decline did not differ significantly between SMA types (χ^2^(_4_) = 6.2533, *P* = 0.181). PEF declined with 0.9%, 2.0%, 1.8%, 1.3% and 1.4% per year in SMA type 1c, 2a, 2b, 3a, and 3b respectively (model parameter estimates are shown in Additional File 2).

**Fig. 2:**
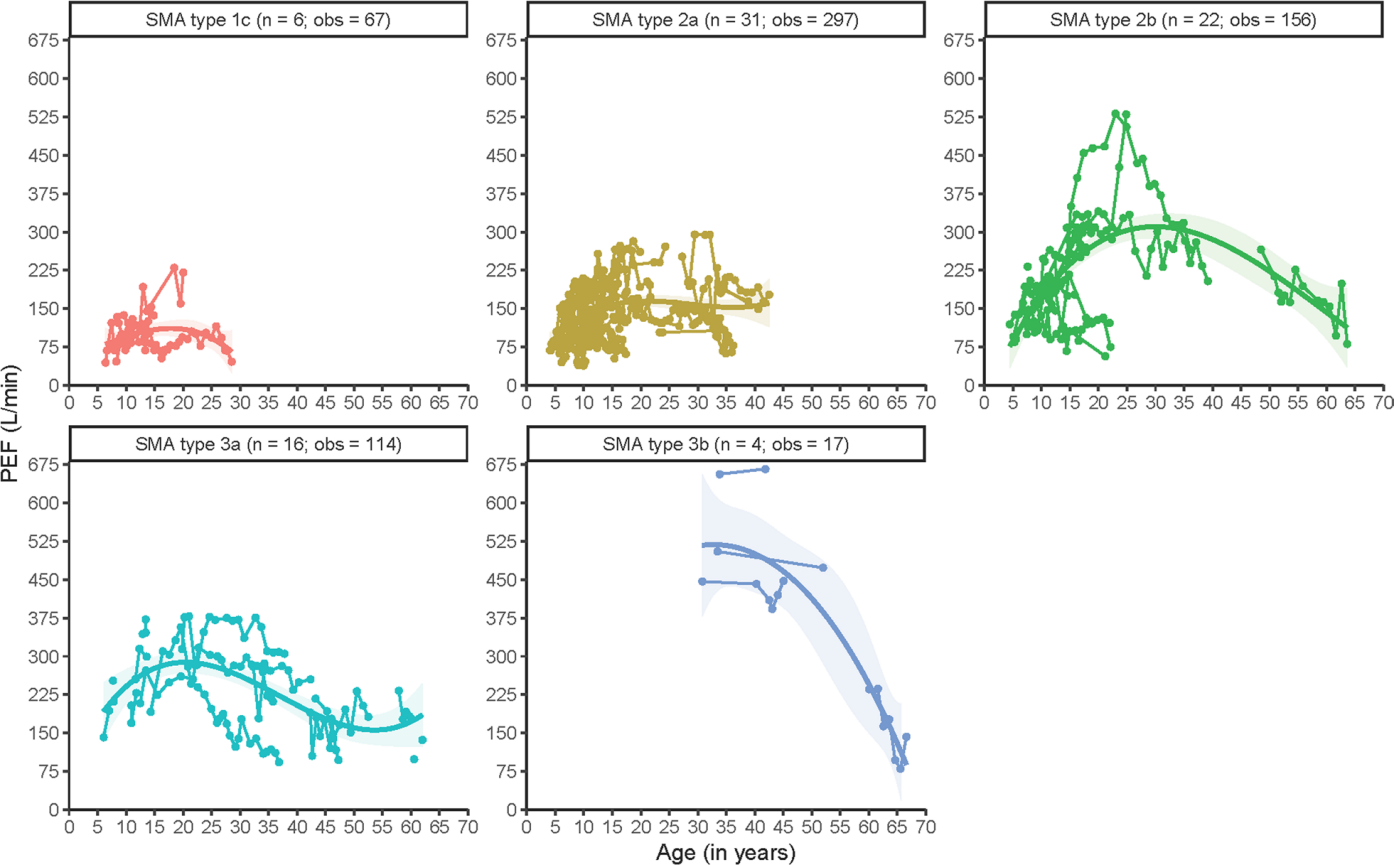
Longitudinal patterns of Peak Expiratory Flow (PEF) in L/min in different SMA types. n = number of patients; obs = number of observations

Non-linear analyses corroborate that PEF decline during early life is largely linear in most SMA types. In SMA type 2a this decline appears to be much faster during early childhood in comparison to children with type 2b. In adults with SMA types 2a, 2b and 3a we observed relative stabilization, although the data suggest that PEF decline can still occur during adulthood. Absolute values of PEF for the different SMA types are shown in Fig. 2.

### Peak cough flow (PCF)

**Fig. 3:**
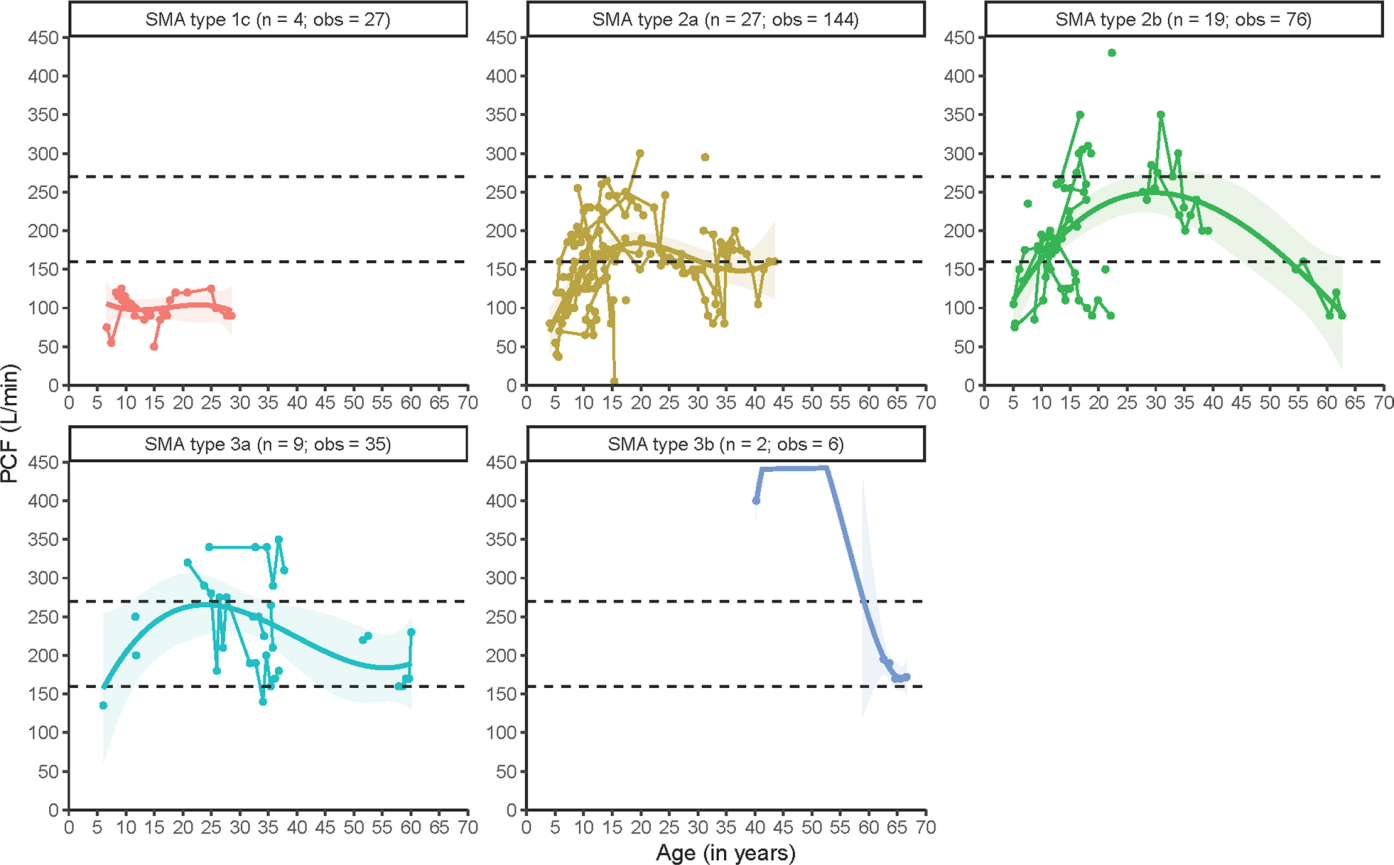
Longitudinal patterns of peak cough flow (PCF) in L/min in different SMA types. n = number of patients; obs = number of observations. The horizontal lines represent two important thresholds. In adults and children over 12 years of age a PCF of 160 L/min is necessary for effective secretion clearance and a PCF of 270 L/min or more is associated with resilience to respiratory infection

We obtained 288 measurements from 61 patients. Longitudinal analyses are shown in Fig. 3, in which the important therapeutic thresholds of 270 L/min [indicating vulnerability to respiratory failure during otherwise trivial respiratory tract infections (RTIs)] and 160 L/min (indicating the boundary below which secretion clearance becomes ineffective) are marked [32].

PCF was lowest in SMA type 1c, with values < 160 L/min throughout life. After early childhood, patients with SMA type 2 reached values between 160 and 270 L/min, with clear differences between types 2a and 2b. Median PCF remained around 160 L/min in type 2a during adolescence and early adulthood, whereas in type 2b median PCF steadily increased until (early) adulthood. Patients with SMA type 3a had higher PCF values from earlier ages onwards in comparison to type 2b, but median values were still well below normal. The limited available data obtained from patients with type 3b indicate that even for these more mildly affected patients, PCF values may decrease in aging individuals.

### Maximal expiratory pressure (PE_max_)

**Fig. 4:**
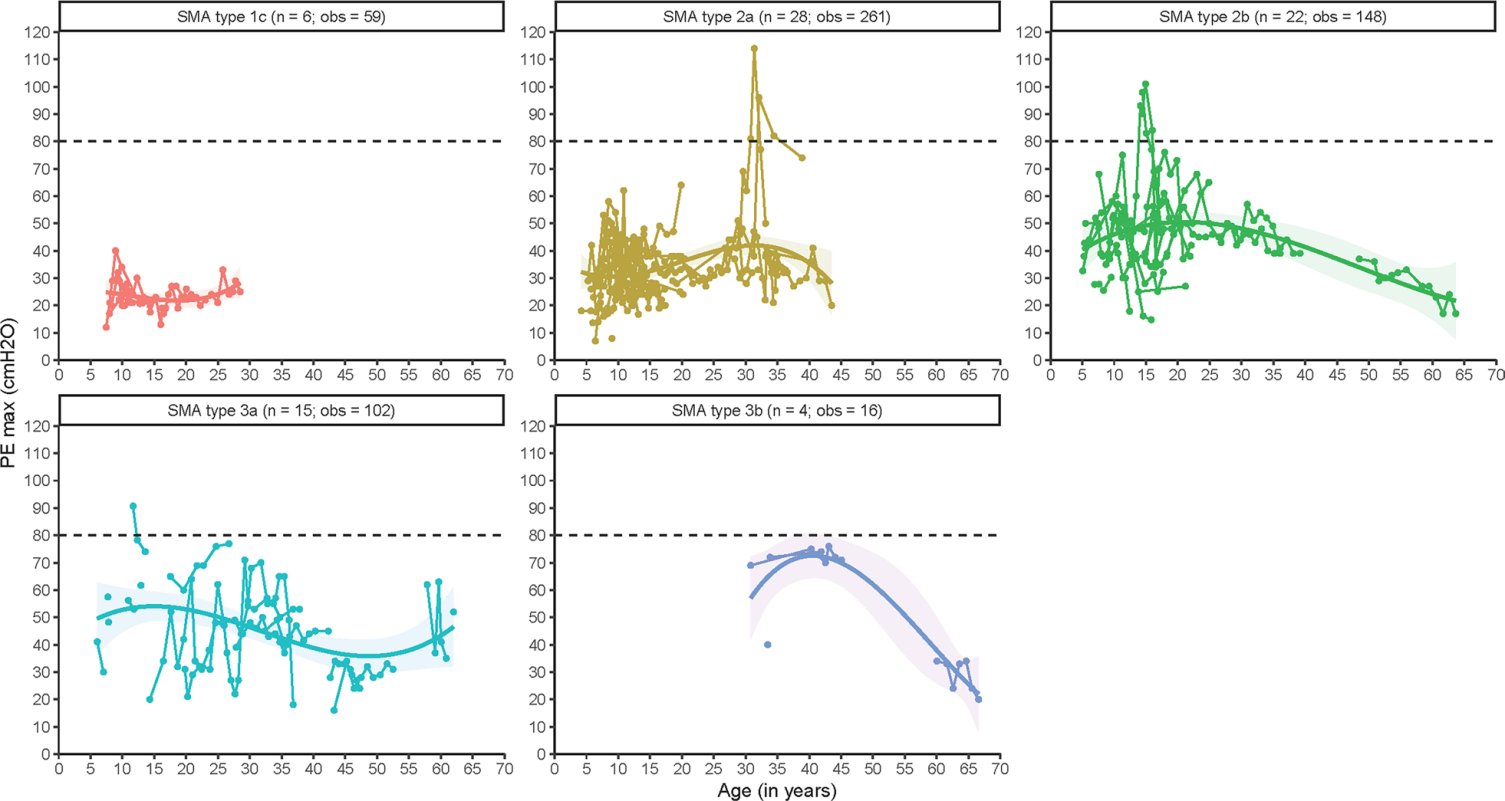
Longitudinal patterns of maximal expiratory pressure (PE_max_) in cmH_2_O in different SMA types. n = number of patients; obs = number of observations. The horizontal line represents the lower limit of normal PE_max_

We analyzed 586 measurements from 75 patients (Fig. 4), showing lower PE_max_ values from early childhood onwards in patients with SMA types 1c–3a compared to the reference population, where PE_max_ values are usually ≥ 80 cmH_2_O during adulthood [20]. Patients with type 1c had severely lowered PE_max_, without improvements with increasing age. PE_max_ in types 2a and 2b increased in adolescence to 40–50 cmH_2_O. It is noteworthy that, despite limited data, all PE_max_ values from patients with SMA type 3b were < 80 cmH_2_O and suggestive of a decline later in life.

### Maximal inspiratory pressure (PI_max_)

**Fig. 5:**
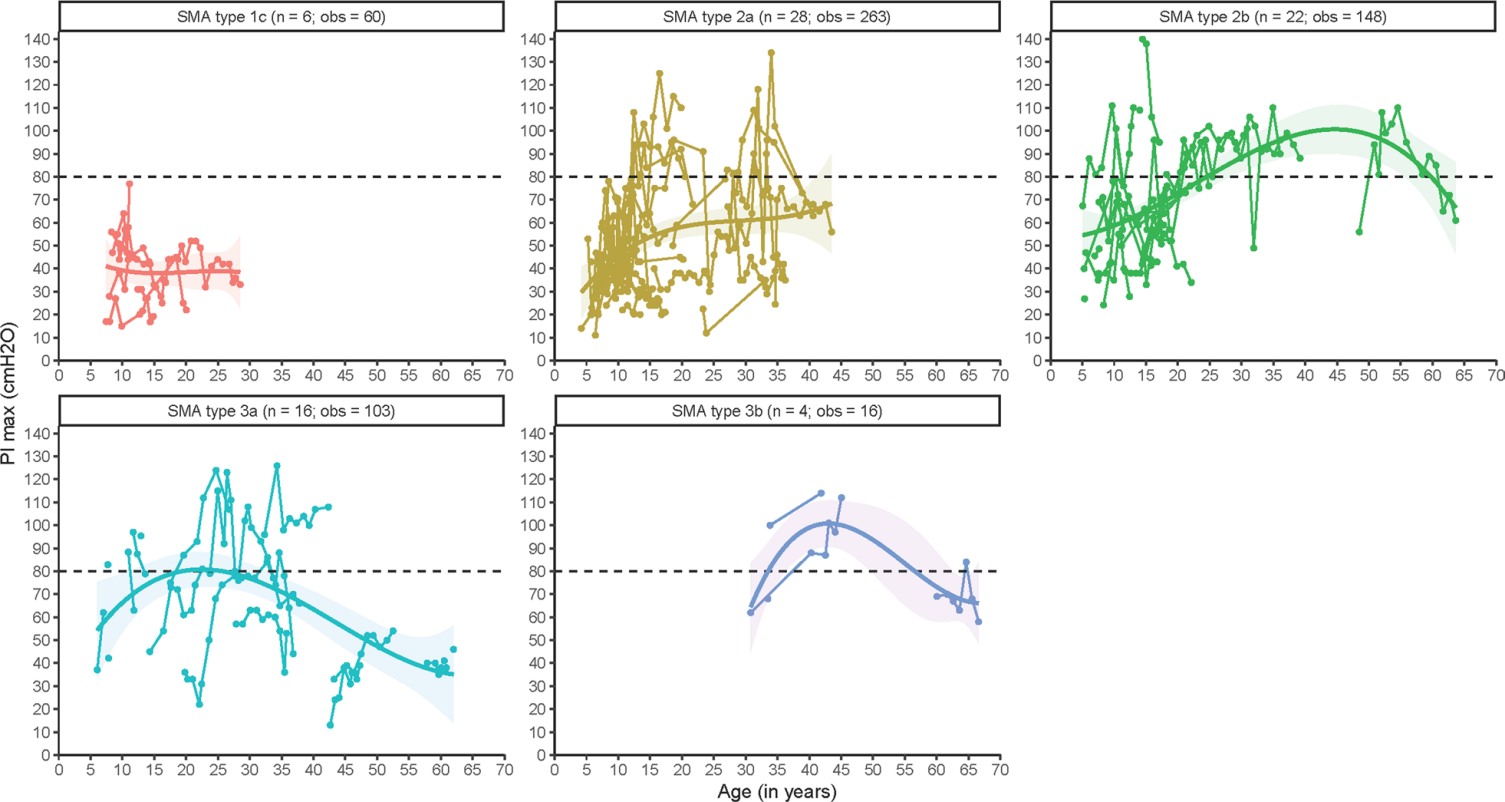
Longitudinal patterns of maximal inspiratory pressure (PI_max_) in cmH_2_O in different SMA types. n = number of patients; obs = number of observations. The horizontal line represents the lower limit of normal PI_max_

We assessed PI_max_ longitudinally using 590 measurements from 76 patients (Fig. 5). Large intra- and inter-individual differences were present, in accordance with findings in the reference population [33]. Overall, PI_max_ was most affected in type 1c without improvements with increasing age. In patients with type 2a, PI_max_ increased to approximately 50–60 cmH_2_O in adolescence. By contrast, patients with SMA type 2b reached PI_max_ values > 80 cmH_2_O during adulthood. Patients with type 3a had a similar pattern, although in our cohort they did decline well below 80 cmH_2_O from approximately 30 years onwards. The limited number of observations precludes definite conclusions for SMA type 3b.

### PE_max_/PI_max_ ratio

**Fig. 6:**
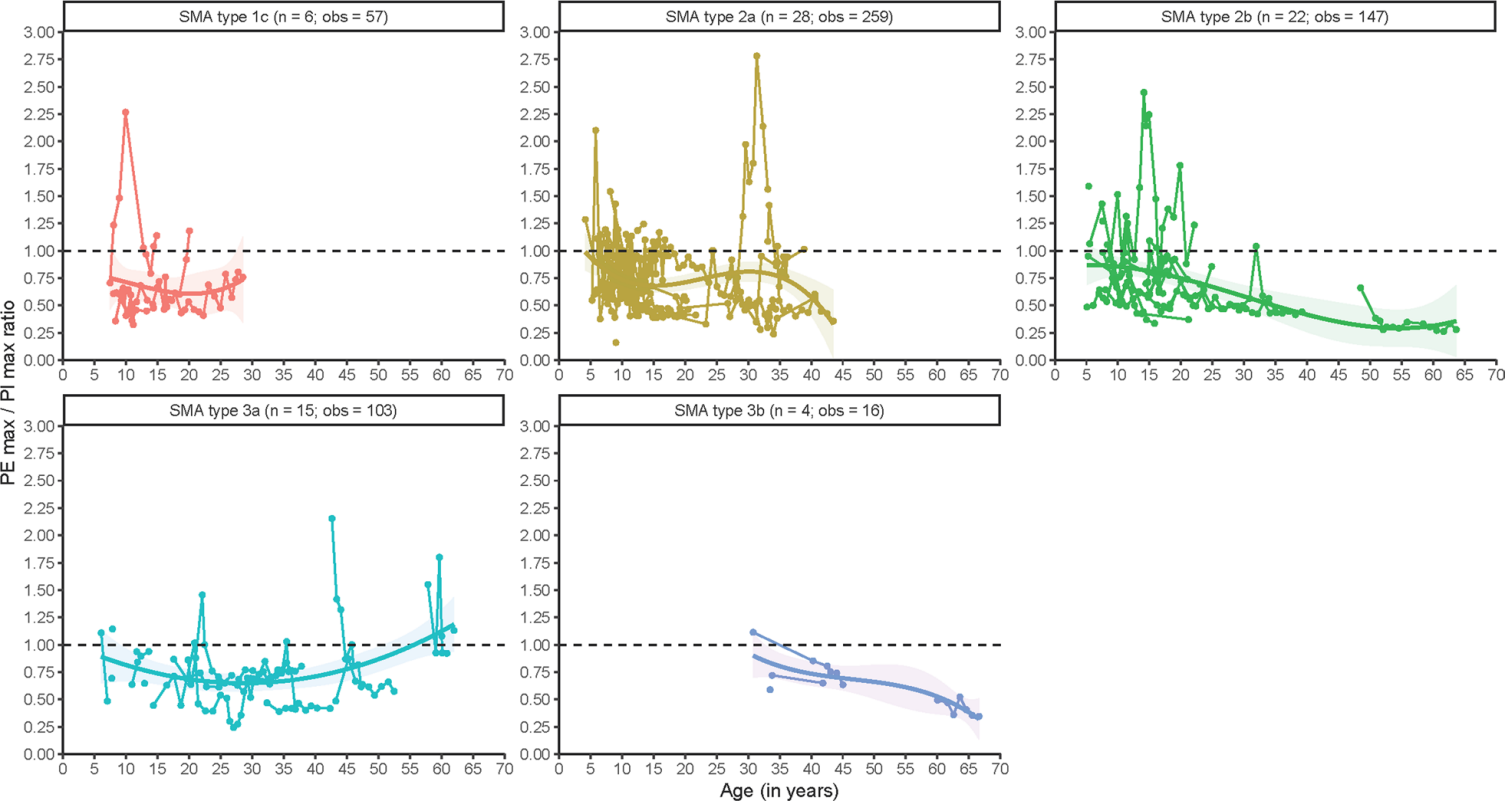
Longitudinal patterns of PE_max_/PI_max_-ratio. n = number of patients; obs = number of observations

We obtained 582 measurements from 75 patients. Figure 6 summarizes the longitudinal course, with a median ratio < 1 for all SMA types, except for a small number of older patients with SMA type 3a (but not type 3b).

### Sniff nasal inspiratory pressure (SNIP)

**Fig. 7:**
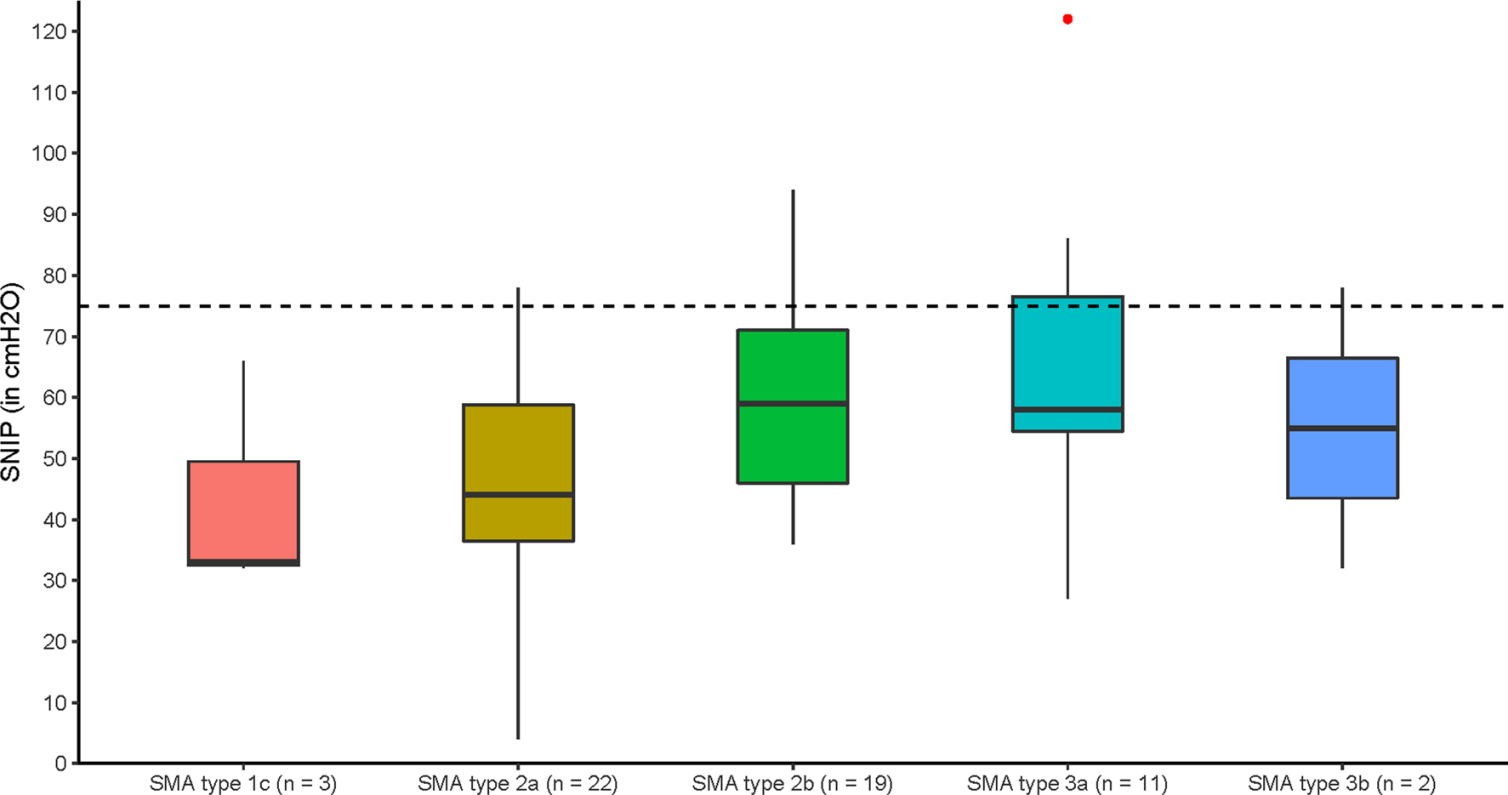
Sniff nasal inspiratory pressure (SNIP) for the different SMA types. Boxplot of median SNIP values for each SMA type; red dots indicate outliers

We used the available SNIP data from 57 patients [median age: 12.9 years (IQR 9.9–29.0)]. SNIP was not statistically different between SMA types (F(4,52) = 2.219, *P* = 0.080). Median SNIP was 33, 44, 59, 58 and 55 cmH_2_O in SMA types 1c, 2a, 2b, 3a, and 3b, respectively. We found a significant trend of increasing SNIP values with milder types (JT = 743, *P* = 0.0053). Importantly, virtually all SNIP outcomes were below 75 cmH_2_O, which is considered the lower limit of normal (Fig. 7).

## Discussion

Here, we present natural history data on the longitudinal course of respiratory muscle strength in treatment-naïve patients with SMA. We show that there are clear differences in respiratory muscle strength between SMA types with a progressive decline. In general, measurements of respiratory muscle strength are most affected in the more severe SMA types. Based upon our data, particularly PEF may be a suitable outcome measure for follow-up of patients with SMA.

Progressive respiratory muscle weakness is the most important cause of morbidity and mortality in patients with SMA [1, 2] and contributes to the increasing dependency on mechanical ventilation of patients with SMA types 1 and 2 [34]. The absence of respiratory function measures as a primary outcome in the pivotal clinical trials of recently introduced genetic therapies for SMA is at least partially explained by the scarcity of reference data [4, 5]. Recent studies on the effect of nusinersen treatment in adult patients have focussed on motor scores and indicate that identification of the ‘ideal’ outcome parameter, reflecting both worsening or improvement in motor function at all grades of disease severity, might not be feasible. This has contributed to the advice that future studies should focus on the long-term effect of nusinersen on other motor-related functions such as ventilation [35]. We recently published a large body of natural history data on lung function in SMA [8], but these spirometry endpoints may be affected by factors that are independent of respiratory muscle dysfunction [36]. Respiratory muscle strength may be an even more appropriate outcome measure [36].

PE_max_ and PI_max_ were severely affected in SMA types 1c–3a. PE_max_ may be the most suitable outcome of these two, as expiratory muscle function is predominantly affected in patients with SMA [9, 10]. Interestingly, PE_max_ was low in patients with SMA type 3a from early ages on, whilst we have previously shown that lung volumes in these patients remain normal at least until (early) adulthood [8]. Based on these data, we believe PE_max_ is a sensitive screening parameter to detect respiratory muscle weakness in SMA patients.

Our findings corroborate the results of some previous cross-sectional studies indicating decreased PE_max_ and PI_max_ with normal lung volumes in patients with SMA types 2 and 3 [15, 21], although different results in two other small studies have also been reported [9, 37].

SNIP has been proposed as an alternative or complementary test to PI_max_. It measures inspiratory strength and normal values exclude inspiratory muscle weakness [32, 38]. In our cohort, SNIP was abnormally lowered in virtually all patients without significant differences between SMA types, although a trend of decreasing SNIP values with more severe phenotypes was present. Our observations are in accordance with the recent work of Kapur [37]. Although SNIP is easy to perform, it may underestimate inspiratory muscle strength in case of nasal obstruction or severe respiratory muscle weakness [38], which may be present from young ages onwards in patients with SMA types 1 and 2. Even though strong correlations between SNIP, PI_max_ and vital capacity have been shown [28], we believe it may be less suited to discriminate between SMA types or as an outcome measure for longitudinal follow-up.

In the absence of bronchial obstruction, PEF reflects maximal expiratory flow [12, 39]. We observed differences at baseline between SMA types and a rather linear decline of PEF in most types over time, resembling the course of FVC in patients with SMA [8]. As the average annual PEF decline did not differ significantly between SMA types, SMA types are primarily separated by differences already present at baseline or occurring very early in life. The observed pattern of relative stabilization in adults with SMA types 2 and 3a could be caused by relative disease stabilization, but we believe it is more likely the consequence of either a floor effect due to difficulties with quantification of very low PEF values or loss to follow-up of most severely affected patients due to death or initiation of invasive mechanical ventilation. Based upon our findings, PEF may be used as an outcome measure for SMA in future studies, as has also been suggested for Duchenne Muscular Dystrophy [40].

Coughing is essential for airway clearance and requires coordinated use of both inspiratory and expiratory muscles, which can be assessed by PCF [28]. PCF in SMA patients had previously only been studied in small cohorts [37]. In our study, nearly all patients had a PCF < 270 L/min. In SMA type 1c and a large number of patients with type 2 PCF was even < 160 L/min. Since low PCF is associated with an increased occurrence of RTIs, PCF could represent a clinically meaningful endpoint for trials.

Our work has important strengths and expands the scarce natural history data on respiratory strength in patients with SMA. First, we investigated a range of measurements reflecting respiratory muscle strength in a large population-based cohort, covering a broad spectrum of SMA severity and a wide age range. Secondly, the large cohort allowed for analyses to assess differences between SMA types. We studied several tests of respiratory muscle weakness as it is known that combining these tests increases diagnostic precision [41]. Finally, to overcome the risk of including inaccurate data from weaker patients, especially young children, professionals experienced in performing these tests in pediatric and adult patients with NMDs conducted all tests.

The generally broad confidence intervals around both intercepts and slopes are a limitation of our work. It reflects the uncertainty of the predicted longitudinal patterns. This can partly be explained by the inability of young children to reliably perform these tests, but also the limited number of observations at older ages for some of the SMA types. The limited number of elderly patients in our cohort is possibly partly explained by SMA-related death or loss to follow-up. However, we do not believe that this changes our conclusion that the general pattern of respiratory muscle strength is one of decline over time. Finally, our study lacks an assessment of possible confounders, such as severity of (corrected) scoliosis, use of airway clearance techniques, or mechanical ventilation. However, we consider this less important as our study focuses on the natural history of SMA with treatment according to the standards of care [1, 6].

## Conclusion

There are clear differences in respiratory muscle strength and its progressive decline between SMA types. In general, measurements of respiratory muscle strength are most affected in the more severe SMA types. PEF declines in a rather linear pattern in all SMA types and is among the most suitable measures to be used for the longer-term follow up of patients and treatment efficacy assessments.

## Supplementary Information

**Additional file 1** Classification of SMA types.

**Additional file 2** Standardized PEF (in %) stratified by SMA type: model parameters estimates.

## Data Availability

The presented model summary statistics allow full reproduction of all LMMs. Additional data supporting our findings are available upon reasonable request.
