# Supplementary material for: Natural history of respiratory muscle strength in spinal muscular atrophy: a prospective national cohort study
Source: Orphanet J Rare Dis. 2022 Feb 21;17:70. doi: 10.1186/s13023-022-02227-7 (PMC8862532; doi:10.1186/s13023-022-02227-7)
Supplement: Supplementary file 1 — Additional file 1 Classification of SMA types. [file 13023_2022_2227_MOESM1_ESM.docx]

**Additional file 1:** Classification of SMA types

| **SMA type** | **Age at symptom onset** | **Highest achieved motor milestone** |
| --- | --- | --- |
| 1 | 0-6 months | Never acquires ability to sit unsupported |
| 0/1a | Prenatal/neonatal | No head control |
| 1b (classic SMA) | 1-6 months | No head controle, unable to roll over |
| 1c | 3-6 months | Some additional motor skills, like head |
|  |  | control or rolling over |
| 2 | 6-18 months | Able to sit unsupported, unable to walk |
| 2a |  | Unsupported sitting, able to stand |
|  |  | or walk with help |
| 2b |  | Unsupported sitting, able to stand or |
|  |  | walk a few steps with help |
| 3 | > 18 months | Able to walk unsupported |
| 3a | 18-36 months |  |
| 3b | > 36 months |  |
| 4 | ≥ 18 years | Able to walk unsupported |
