# Supplementary material for: Natural history of respiratory muscle strength in spinal muscular atrophy: a prospective national cohort study
Source: Orphanet J Rare Dis. 2022 Feb 21;17:70. doi: 10.1186/s13023-022-02227-7 (PMC8862532; doi:10.1186/s13023-022-02227-7)
Supplement: Supplementary file 2 — Additional file 2 Standardized PEF (in %) stratified by SMA type: model parameters estimates. [file 13023_2022_2227_MOESM2_ESM.docx]

**Additional File 2:**

Standardized PEF (in %) stratified by SMA type: model parameters estimates

|  |  |  | Fixed effects |  |  | Random effects | |
| --- | --- | --- | --- | --- | --- | --- | --- |
| **SMA type** | n | Intercept (SE) | 95% CI intercept | Slope | 95%CI slope | SD intercept | SD slope |
| 1c | 6 | 49.30 (4.52) | 41.41; 57.92 | -0.89 | -1.71; 0.14 | 3.58 | 0.64 |
| 2a | 31 | 72.52 (5.10) | 62.18 ; 82.56 | -2.04 | -2.73; -1.37 | 24.76 | 1.46 |
| 2b | 22 | 86.90 (5.67) | 74.99; 98.21 | -1.82 | -2.63; -1.05 | 18.32 | 1.12 |
| 3a | 16 | 95.86 (7.55) | 79.91; 110.68 | -1.31 | -1.80; -0.87 | 24.06 | 0.65 |

*Legend:* CI = confidence interval; *n* = number of patients in each group; SD= standard deviation; SE = standard error.
